# A classification method for soybean leaf diseases based on an improved ConvNeXt model

**DOI:** 10.1038/s41598-023-46492-3

**Published:** 2023-11-06

**Authors:** Qinghai Wu, Xiao Ma, Haifeng Liu, Cunguang Bi, Helong Yu, Meijing Liang, Jicheng Zhang, Qi Li, You Tang, Guanshi Ye

**Affiliations:** 1 Electrical and Information Engineering College, Jilin Agricultural Science and Technology University, Jilin, 132101 Jilin China; 2 School of Information and Control Engineering, Jilin Institute of Chemical Technology, Jilin, 132022 China; 3 College of Agriculture, Yanbian University, Yanji, 133002 Jilin China; 4 College of Information Technology, Jilin Agricultural University, Changchun, 132101 Jilin China; 5 Department of Crop and Soil Sciences, Washington State University, Pullman, WA 99164 USA; 6 College of Electronic and Information, Northeast Agricultural University, Harbin, Heilong, 150030 Jiang China

**Keywords:** Machine learning, Classification and taxonomy

## Abstract

Deep learning technologies have enabled the development of a variety of deep learning models that can be used to detect plant leaf diseases. However, their use in the identification of soybean leaf diseases is currently limited and mostly based on machine learning methods. In this investigation an enhanced deep learning network model was developed to recognize soybean leaf diseases more accurately. The improved network model consists of three parts: feature extraction, attention calculation, and classification. The dataset used was first diversified through data augmentation operations such as random masking to enhance network robustness. An attention module was then used to generate feature maps at various depths. This increased the network’s focus on discriminative features, reduced background noise, and enabled the use of the LeakyReLu activation function in the attention module to prevent situations in which neurons fail to learn when the input is negative. Finally, the extracted features were then integrated using a fully connected layer, and the predicted disease category inferred to improve the classification accuracy of soybean leaf diseases. The average recognition accuracy of the improved network model for soybean leaf diseases was 85.42% both higher than the six deep learning comparison models (ConvNeXt (66.41%), ResNet50 (72.22%), Swin Transformer (77.00%), MobileNetV3 (67.27%), ShuffleNetV2 (59.89%), and SqueezeNet (72.92%)), thus proving the effectiveness of the improved method.The model proposed in this paper was also tested on the grapevine leaf dataset, and the performance ability of the improved network model remained due to other common network models, and overall the proposed network model was very effective in leaf disease identification.

## Introduction

Soybean is one of the most widely cultivated legumes, as it can be processed into various foods and is an important global feed crop^1^. Disease is one of the main factors affecting soybean yields, with infected soybeans generally reducing yields by 10–30%, or more than 50% in severe cases. In particular, soybean leaf diseases can alter leaf color and induce leaf loss which when severe can lower their disease resistance, thus decreasing crop yield and quality. Common and serious leaf diseases include soybean leaf spots and soybean rust. According to treatment plans, appropriate actions should be taken at the earliest possible stages of disease occurrence^2^. It is thus essential that we detect such diseases in a timely and accurate manner. Traditional disease identification requires manual intervention, and due to the complexity and diversity of leaf diseases, a great deal of experience is required to accurately identify the disease type, which makes it a time-consuming and labor-intensive process^3^. Numerous algorithms have been used to identify plant leaf diseases owing to the development of machine learning and neural networks^4^. Currently, there are two primary approaches for plant leaf disease classification tasks: leaf disease recognition based on traditional machine learning algorithms and disease recognition based on deep learning.

Feature extraction and classification based on conventional machine learning algorithms is one of the traditional methods used to categorize plant leaf diseases. The benefits of this approach include a quick recognition speed and minimal hardware requirements. Panigrahi et al.^5^ compared and analyzed traditional machine learning algorithms such as naïve Bayes (NB), decision tree (DT), K-nearest neighbor (KNN), support vector machine (SVM), and random forest (RF) for maize disease detection. Random forest had the highest accuracy when compared to the other algorithms. Rahaman et al.^6^ proposed a k-nearest neighbor (KNN) classifier to detect and classify plant leaf diseases using texture features extracted from disease images, which was successful in detecting and identifying the selected diseases with 96.76% accuracy. The general steps of image classification using machine-learning algorithms are image preprocessing, feature extraction, and classifier training. Among these, feature extraction is the most crucial step as it directly impacts on the accuracy of the classification result. Traditional feature extraction relies mainly on manual design, which can make feature extraction challenging^7^.

With the development of neural networks and deep learning^8^ researchers have shifted their focus away from automated feature extraction using neural networks. Lin et al.^9^ proposed the GrapeNet lightweight model to identify the different stages of grape leaf disease, which addresses the problem of diverse disease morphologies and leads to increased recognition accuracy and reduced parameter quantity. Bansal et al.^10^ combined three pre-trained models to identify apple tree leaves and achieved satisfactory classification results. Haque et al.^11–14^ conducted experiments on common maize diseases, using GoogLeNet, Inception V3, and designing a new CNN network to classify and assess the severity of common maize diseases, respectively, and the proposed methods achieved good results in the identification of maize leaf diseases, and achieved certain results in the lightweighting of the model.

To date, however, studies on soybean leaf disease recognition have been limited^15–17^, with the majority focusing on the images of soybean leaf disease captured against a single background, and mostly using machine learning methods. In this study, soybean leaf images with complex background information were used as the training, validation, and testing samples, and an improved ConvNeXt model-based soybean leaf disease image classification algorithm was proposed that addresses the issue of complex background interference typically present in real collections. The main findings of this study can be summarized as follows: (1) by performing image augmentation operations, such as random masking, on the dataset before training the network, the dataset was enriched to improve model robustness; (2) attention mechanisms were included at various depths to enable the ConvNeXt network to focus on discriminative features and reduce background interference; (3) the LeakyReLU activation function was used in the attention module to avoid situations in which the neurons did not learn when the input was negative; and (4) the improved network model was compared to existing models under the same conditions to validate its disease classification ability.

## Materials and method

### Data augmentation

The experiment was conducted to investigate soybean leaf spot, soybean rust, and healthy leaves. Soybean leaf spot disease initially appears as watery spots on the leaves, then turns brown or black with a yellow-green halo around the edges. Soybean rust is caused by the phakopsora pachyrhiz and usually spreads upward from the lower leaves, with small yellow spots appearing on the leaves in the early stages of the disease, and then the spots expand slightly. Pictures displaying each class of disease are shown in Table 1.

**Table 1:**
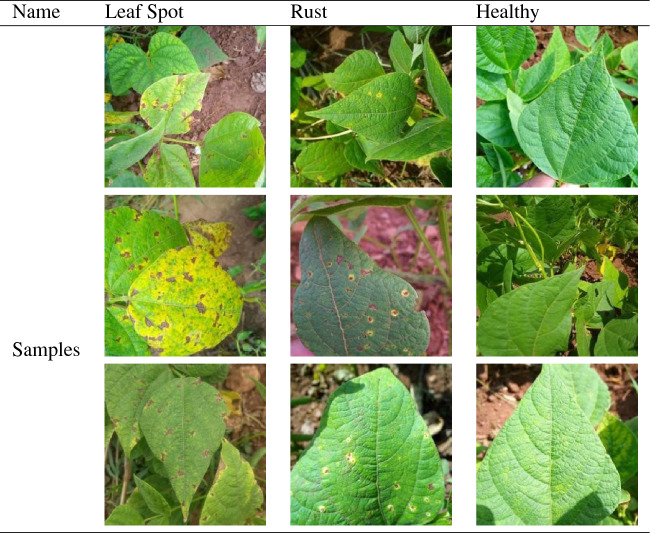
Samples of soybean leaf disease.

**Figure 1:**
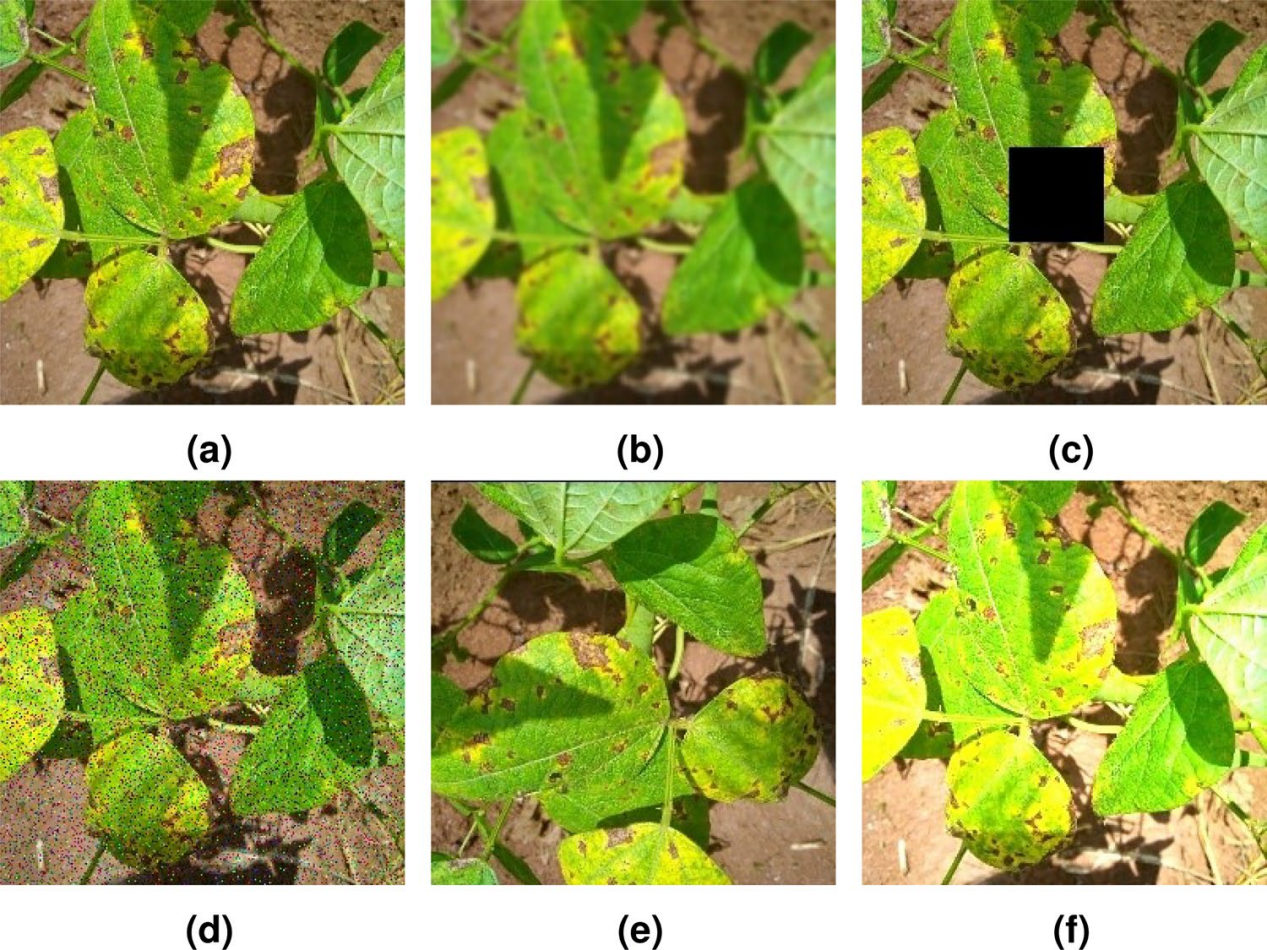
Data enhancement effect. (**a**) Original images, (**b**) Gaussian blur, (**c**) Random occlusion, (**d**) Add noise, (**e**) Rotate, (**f**) Brightness adjustment.

Due to the variations in weather conditions and leaf-covered lesions identified when collecting disease images, the generalization ability and robustness of the model were poor during soybean disease classification. To improve the classification accuracy of the model , as well as its generalization ability and robustness, a regularization method for image data augmentation is typically used^18^. To simulate the different angles, background leaf occlusions, and weather conditions encountered during image acquisition, this study used data augmentation methods such as rotation, Gaussian blur, adding random noise, adding random occlusion at random positions, and brightness adjustment. This prevented overfitting and improved the robustness and generalization of the model. The “Smart Agriculture” platform dataset from Jilin Agricultural Science and Technology College (Collected from soybean plantations at Jilin College of Agricultural Science and Technology, Jilin Province, China) was used as the experimental objects. The original dataset was split into training, validation, and testing sets using a ratio of 6:2:2 to prevent information leakage. A total of 1296 images were used before image augmentation, and 11655 images were used after data augmentation. Detailed dataset information is presented in Table 2, and typical data augmentation effects are shown in Fig. 1.

**Table 2:** Details of the soybean leaf disease dataset.

Name	Original images	After data enhancement
Leaf spot	432	3,888
Rust	436	3,924
Healthy	428	3,843
Total	1,296	11,655

### Attention mechanism

The human visual system serves as the inspiration for the attention mechanism as attention can quickly be focused on the key elements of a scene , allocating more neural network computational resources to critical tasks when processing complex information^19^. By using backpropagation to guide the attention module, important features can be identified using parameter updates, enabling efficient and accurate task completion. Attention mechanisms have been widely used in a variety of fields^20–23^.

**Figure 2:**
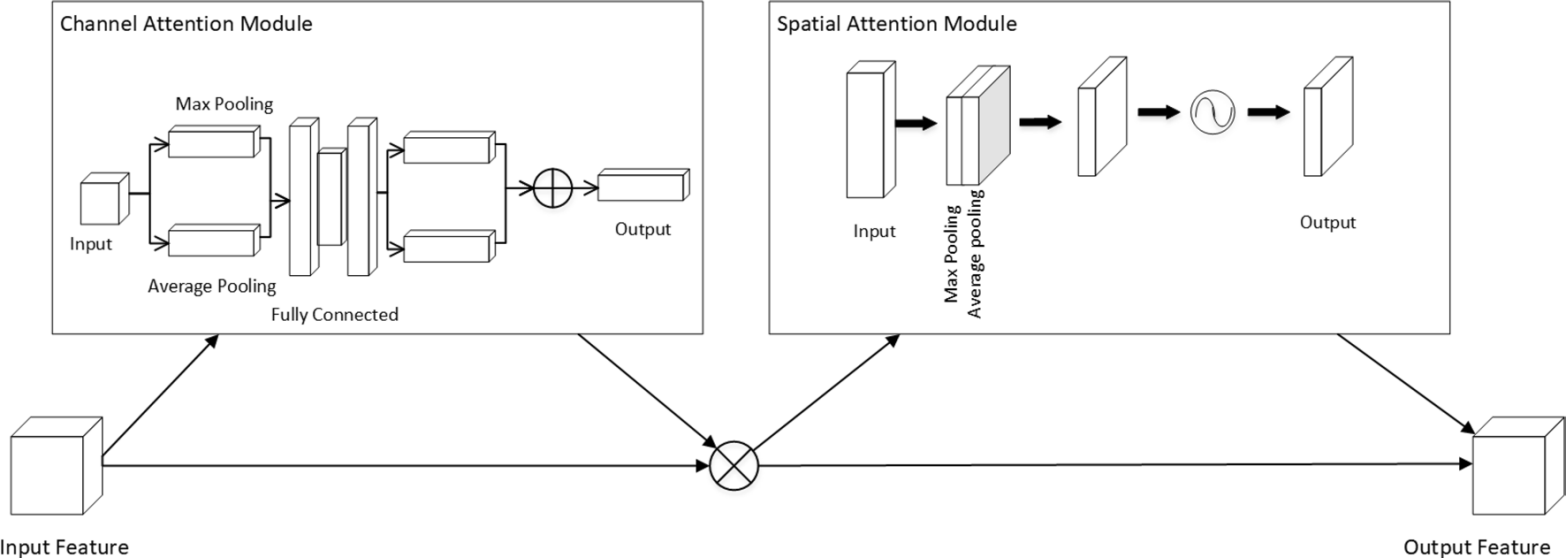
CBAM monolithic construction.

Common attention mechanisms include Squeeze and excitation (SE-Net), efficient channel attention (ECA-Net), and the convolutional block attention modules (CBAM). SE-Net^24^ explicitly models the interdependencies between feature channels; it automatically acquires the importance of each channel through learning. ECA-Net^25^ proposes a non-diminished local cross-channel interaction strategy and an adaptive method for selecting one-dimensional convolution kernel size. CBAM^26^ is a combined spatial and channel attention mechanism module, which can well obtain the attention information between space and channel, it includes two parts: spatial attention module (SAM) and channel attention module (CAM), which are used to collect the attention information from both space and channel respectively. It is this attentional mechanism that is used in this study. CBAM’s overall structure of the CBAM is shown in Fig. 2. The computation formula of the channel attention module (CAM) is shown in Eq. 1:

1 $$\begin{aligned} CAM(F)=\sigma ({{W}_{1}}({{W}_{0}}(F_{avg}^{c}))+{{W}_{1}}({{W}_{0}}(F_{\max }^{c}))) \end{aligned}$$

where $\sigma$ describes the sigmoid activation function, $_{^{{{F}_{avg}}}}$ and $_{^{{{F}_{max}}}}$ describe the output results of global average pooling and global maximum pooling, respectively, and ${{W}_{0}}$ and ${{W}_{1}}$ describe two different neural network operations in two layers.

The computation formula of the spatial attention module (SAM) is shown in Eq. 2:

2 $$\begin{aligned} SAM(F)=\sigma \Big (f^{7\times 7}\big (F_{avg}^{s};F_{max}^{s}\big ) \Big ) \end{aligned}$$

where ${{f}^{7\times 7}}$ describes the convolution operation with a kernel size of 7$\times$7, and [] describes the channel concatenation operation.

The overall CBAM process can be described by Eqs. 3 and 4:

3 $$\begin{aligned} {F}'&=CAM(F)\otimes F \end{aligned}$$

4 $$\begin{aligned} {F}''&=SAM({F}')\otimes {F}' \end{aligned}$$

where the input feature is $F(F\in \{{{R}^{C\times H\times W}}\})$, the output of the channel attention module is $CAM(CAM\in \{{{R}^{C\times 1\times 1}}\})$ , the output of the spatial attention module is $SAM(SAM\in \{{{R}^{1\times H\times W}}\})$, and the output results of the channel and spatial attention are denoted as ${F}'$ and ${F}''$ respectively. This study uses an attention module that combines channel and spatial attention.

### Improved ConvNeXt architecture

**Figure 3:**
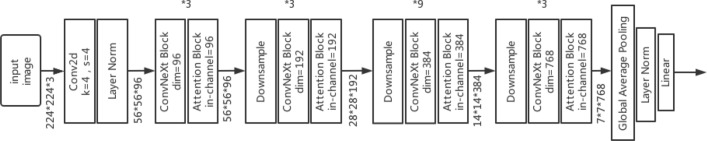
Overall structure of the CBAM-ConvNeXt.

**Figure 4:**
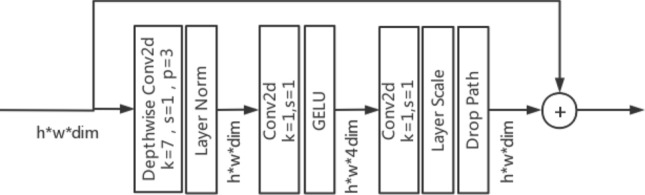
ConvNeXt Block structure.

The ConvNeXt^27^ model used in this experiment was based on ResNet50^28^ and was improved by integrating the ideas of the Swin Transformer^29^. The improved ConvNeXt (CBAM-ConvNeXt) structure proposed in this study (Fig. 3) consists of ConvNeXt Block modules for feature extraction (Fig. 4), downsampling modules, and attention modules to eliminate complex background interference. Typical attention mechanisms include SE-Net, ECA-Net, selective kernel networks (SK-Net)^30^ and CBAM. In this experiment, a comparative analysis was conducted using the SENet, ECANet, SKNet, and CBAM attention modules. CBAM was selected as the attention module. CBAM is an attention mechanism that combines channel and spatial attention. It maps the extracted intermediate features to the channel and spatial dimensions for attention analysis. The obtained attention scores are multiplied by the input of intermediate feature maps to obtain feature maps with added attention, which are then processed by the next convolution operation.

The LeakyReLU^31^ activation function was used in the CBAM attention module to overcome the problem in which neurons do not learn when the input is negative by correcting its nonlinear unit. The LeakyReLU activation function formula is shown in Eq. 5:

5 $$\begin{aligned} Leaky{\text {Re}}LU=\left\{ \begin{matrix} x,x>0 \\ ax,x\le 0 \\ \end{matrix} \right. \end{aligned}$$

The improved ConvNeXt model first extracted shallow features from 224$\times$224 three-channel colored soybean leaf disease images using a 4$\times$4 convolutional operation with a stride of four. A 56$\times$56 feature map with 96 channels was created using a normalization layer. The formulae for calculating the width and height of the feature map obtained after the convolutional operation are shown in Eqs. 6 and 7:

6 $$\begin{aligned} H&=(h-k+2p)/s+1 \end{aligned}$$

7 $$\begin{aligned} W&=(w-k+2p)/s+1 \end{aligned}$$

where H and W are the height and width of the feature map after convolution, respectively, h and w are the height and width of the input feature map, respectively, k is the size of the convolution kernel, p is the padding size, and s is the stride of the convolution. Next, four ConvNeXt blocks, four attention blocks, and three downsampling modules were used to extract features, add attention scores, and downsample feature maps, enabling the network to focus on disease features and reduce its attention on the complex backgrounds, thereby reducing interference.

The softmax function was used as the output of the model to calculate the predicted class of the target. The definition of softmax is given in Eq 8:

8 $$\begin{aligned} Y(P)=P(y=p|x,{{\theta }_{p}})=\frac{{{e}^{{{x}^{T}}\cdot {{\theta }_{p}}}}}{\sum \nolimits _{p=1}^{c}{{{e}^{{{x}^{T}}\cdot {{\theta }_{p}}}}}} \end{aligned}$$

### Evaluation metrics

The cross-entropy loss function was used as the standard for network optimization, and the Adam optimizer was used to optimize the model parameters. The expression for the cross-entropy loss function is given by Eq. 9:

9 $$\begin{aligned} \begin{matrix} \min \\ \{W,b;\theta \} \\ \end{matrix}\varepsilon (W,b;\theta )=\frac{1}{N}\sum \limits _{n=1}^{N}{\left[ -\sum \limits _{p=1}^{N}{\gamma ({{y}_{n}}=p)\cdot \log P(y=p|x,{{\theta }_{p}})}\right] +\lambda R(\theta )} \end{aligned}$$

where C are the number of categories, N are the number of samples, $\gamma$ are the Dirichlet function, the parameters $\theta =({{\theta }_{1}},{{\theta }_{2}},...,{{\theta }_{k}})$, R (•) are the regularization constraint term, and $\lambda$ are the regularization factor.

This experiment used accuracy, precision, recall, and ${{f}_{1}}-score$ to evaluate model performance. Accuracy describes the overall accuracy of the model’s predictions; however, in cases of imbalanced datasets, it may not be a good metric for evaluating model performance. Precision describes the accuracy of positive samples predicted by the model, whereas recall describes the probability of correctly predicting positive samples among all positive samples. The accuracy, precision, recall, and ${{f}_{1}}-score$ for the binary classifications were defined using Eqs. 10–13:

10 $$\begin{aligned} Accuracy&=\frac{TP+TN}{TP+TN+FP+FN} \end{aligned}$$

11 $$\begin{aligned} Precision&=\frac{TP}{TP+FP} \end{aligned}$$

12 $$\begin{aligned} Recall&=\frac{TP}{TP+FN} \end{aligned}$$

13 $$\begin{aligned} {{f}_{1}}-score=2\times \frac{\Pr ecision\times {\text {Re}}call}{\Pr ecision+{\text {Re}}call} \end{aligned}$$

True positive (TP) describes the number of positive samples predicted correctly, false positive (FP) describes the number of negative samples predicted as positive, true negative (TN) describes the number of negative samples predicted correctly, and false negative (FN) describes the number of positive samples predicted as negative.

A confusion matrix was adopted to display the classification results of the model, where each column describes the predicted label category, and each row describes the true label category of the data. The more concentrated the data are on the diagonal, the better the classification performance of the model.

## Results and discussion

Python 3.7 was used as the programming language, and the PaddlePaddle 2.3.2 deep learning framework, was adopted for this experiment . The training process used an accelerated 4-core CPU and Tesla V100 GPU. The network was trained using cross-entropy loss combined with an Adam optimizer^32^, which can adaptively adjust the learning rate based on the training parameters. The network was trained for 100 iterations with a batch size of 64 and learning rate of 0.000001.

To verify the performance of the improved CBAM-ConvNeXt model, two comparative experiments were conducted: a comparison of the effects of training the improved model with the augmented dataset versus the original dataset, and a performance comparison between the improved model and common traditional classification models. In order to validate the classification ability of the network model proposed in this study on other datasets, this paper also conducts a comparison between the proposed model and other network models on the grape leaf dataset.

### Data augmentation experiment results

**Figure 5:**
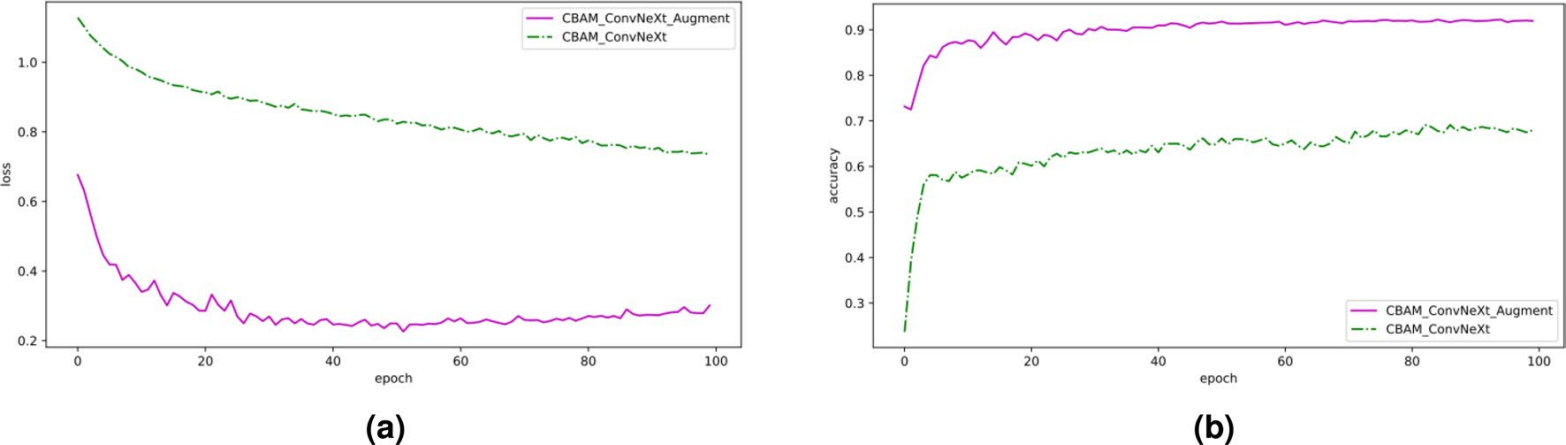
Loss value and accuracy curve before and after data enhancement. (**a**) Loss curves before and after data augmentation, (**b**) Accuracy curves before and after data augmentation.

**Figure 6:**
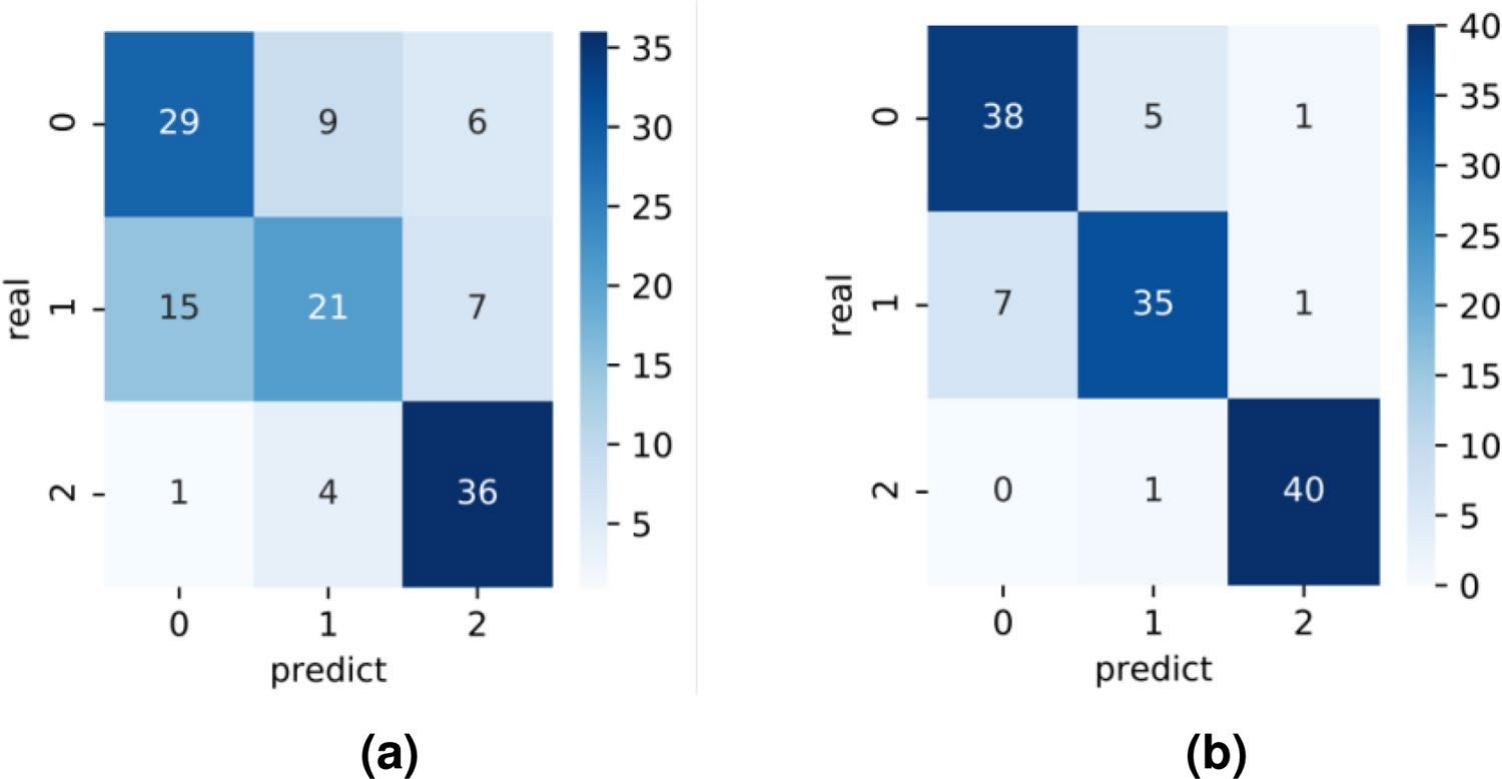
Confusion matrix comparison before and after data enhancement. (**a**) Original dataset, (**b**) After data augmentation.

To verify the improvements in model performance that occur when using the data augmentation method proposed in this paper, the original and augmented datasets were separately fed into the CBAM-ConvNeXt model for training. A comparison of the loss value and accuracy of the validation set during the model training process is shown in Fig. 5. The confusion matrix of the classification performance on the test set is presented in Fig. 6, where larger values and darker colors on the diagonal of the confusion matrix indicate better model classification performance.

The model trained using the augmented dataset was improved in terms of loss values and classification accuracy, both for convergence speed and stable final values, when compared with the model trained using the original dataset (Fig. 9). A comparison of the experimental results before and after data augmentation is presented in Table 3. The data show that the model trained using the augmented dataset had improved accuracy, precision, recall, and f1-score on the test set when compared to the model trained using the original dataset. The improvement of the model’s ability to classify diseases depends on the fact that the data-enhanced training set effectively avoids the generation of overfitting situations during the training process by adding interference information, which improves the robustness of the model. In summary, the data augmentation method used in this study could improve the generalizability and robustness of the network model during the data pre-processing stage, which has a positive effect on the classification of soybean leaf diseases.

**Table 3:** Comparison of experimental effects before and after data enhancement.

Dataset	Accuracy (%)	Precision (%)	Recall (%)	F1-score (%)
Original dataset	67.19	66.56	67.52	66.57
After data augmentation	88.28	88.35	88.44	88.37

### Model improvement experimental results

**Figure 7:**
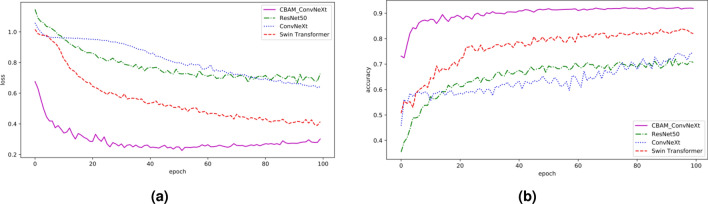
Loss values and accuracy curves for each model. (**a**) Loss curves for each model, (**b**) Accuracy curves for each model.

To verify the effectiveness of the improved model for soybean leaf disease classification, the enhanced dataset was input into the original ConvNeXt, ResNet50, and swine transformer models for training. Comparison curves of the validation loss and accuracy were obtained (Fig. 7). Compared to the other three models, the improved network model showed higher accuracy and loss values and faster convergence due to fuller access to spatial and inter-channel feature information during training. In addition, several common classification models were added as controls. The experimental results for each model are listed in Table 4.

**Table 4:** Comparison of the experimental results of each model.

Models	Accuracy (%)	Precision (%)	Recall (%)	F1-score (%)	Total params	Training time (s)
ResNet50	72.22	72.32	72.26	72.15	23,567,299	7,820.79
ConvNeXt	66.41	65.97	66.52	66.14	27,822,435	9,290.13
Swin transformer	77.00	76.71	77.12	76.48	27,521,661	8,772.59
CBAM-ConvNeXt	85.42	85.53	85.46	85.87	28,162,455	8,707.11
MobileNetV3	67.27	67.12	67.33	67.19	4,230,275	6,682.45
ShuffleNetV2	59.89	59.07	59.97	59.23	1,272,859	6,369.60
SqueezeNet	72.92	72.70	72.98	72.73	736,963	6,761.99

**Figure 8:**
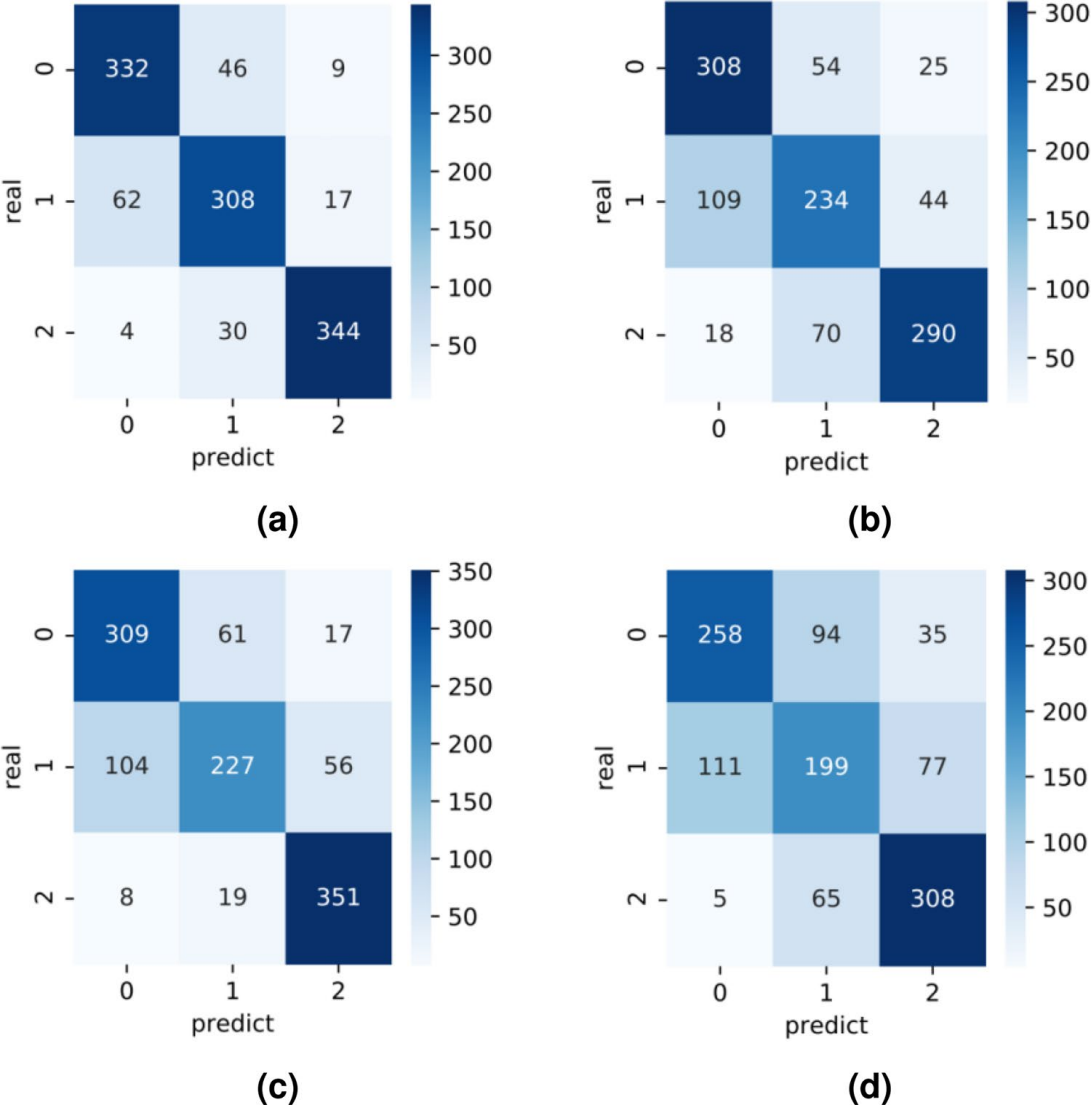
Confusion matrix contrast. (**a**) CBAM-ConvNeXt, (**b**) ResNet50, (**c**) Swin Transformer, (**d**) ConvNeXt.

**Figure 9:**
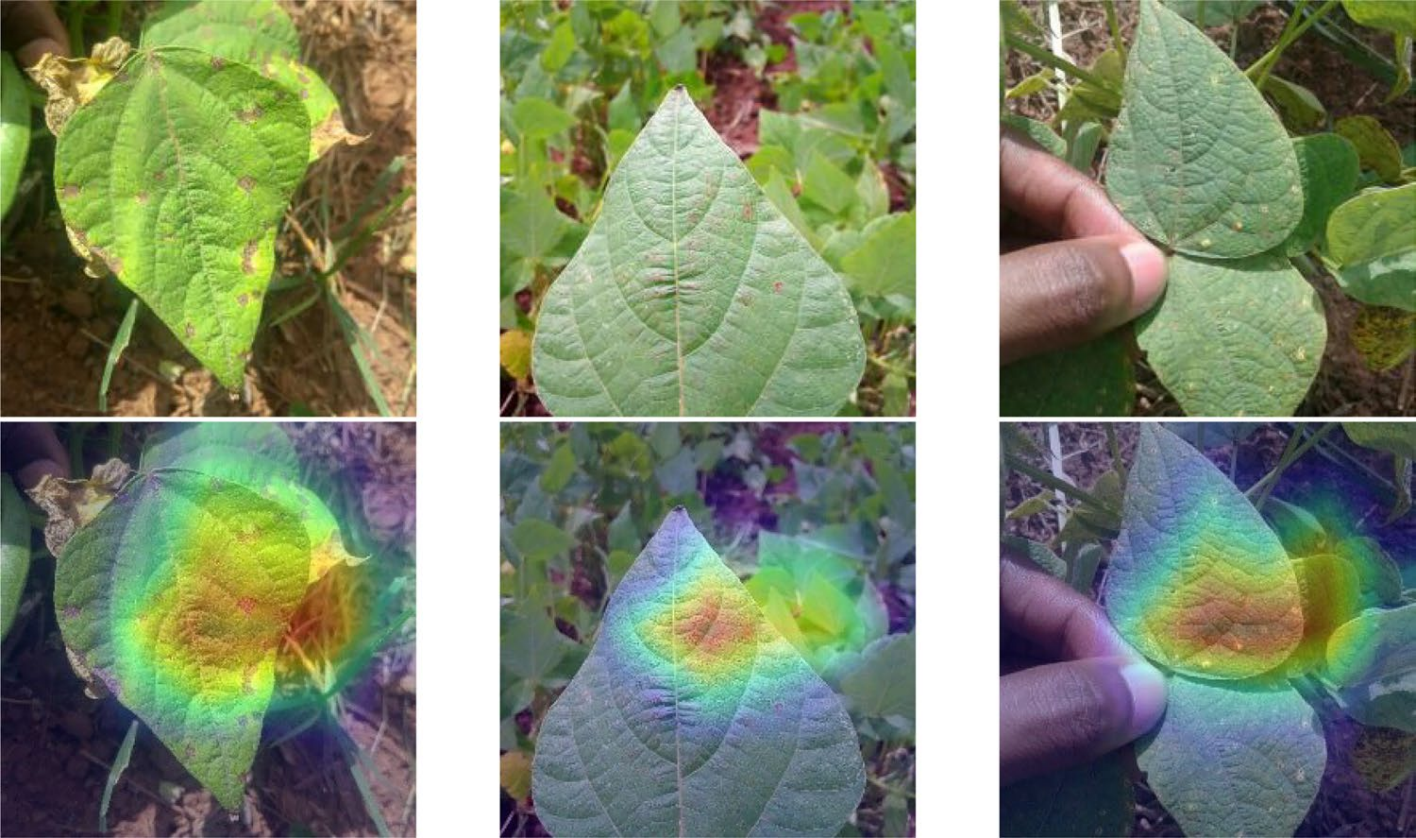
Heat map of attention output. The red and blue regions highlight the most and the least discriminative regions, respectively.

From Table 4, it can be concluded that the proposed model is effective in improving the classification accuracy of the model compared to traditional deep learning classification models in complex contexts with guaranteed parameters and training speed, but lacks in the number of parameters and training speed compared to lightweight network models. The models were then compared using a confusion matrix (Fig. 8). The results showed that misclassifications mostly occurred between classes 0 and 1 because of the high similarity in their lesion features. However, compared to the other three models, the improved ConvNeXt model proposed in this study was superior in identifying and characterizing lesions and their features due to its excellent feature extraction ability to discriminate complex disturbances. A heat map of the output of the last attention module shows that the attention mechanism was effective in the improved network (Fig. 9)^33^.

In addition, to demonstrate the performance of the proposed models on other datasets, we also conducted comparative experiments on the grape leaf dataset in the PlantVillage public dataset, and the experimental results for each model are shown in Table 5.

**Table 5:** Experimental results of the model on the grape leaf dataset.

Models	Accuracy (%)	Precision (%)	Recall (%)	F1-score (%)	training time (s)
ResNet50	87.60	87.50	87.60	87.52	15,495.57
ConvNeXt	85.14	85.13	85.14	84.99	19,618.43
Swin transformer	93.87	93.85	93.87	93.86	21,336.28
CBAM-ConvNeXt	94.52	94.46	94.52	94.47	17,135.20
MobileNetV3	83.56	83.66	83.56	83.59	14,989.98
ShuffleNetV2	78.56	78.79	78.56	78.60	14,905.33
SqueezeNet	87.07	86.87	87.07	86.91	14,070.32

## Conclusion

To address the problem of complex backgrounds in soybean leaf images and unsatisfactory recognition accuracy, a soybean leaf disease classification method based on ConvNeXt and an attention module has been proposed. The proposed method optimizes soybean leaf disease classification by obtaining attention feature maps at different depths in the network. The major conclusions were as follows: By incorporating four CBAM attention modules, the CBAM-ConvNeXt network described in this study was an improvement when compared with the original ConvNeXt model as it enhanced the attention of the network to the interchannel and spatial positions of the feature maps. The issue of neuronal inactivity when the input was negative was addressed by implementing the LeakyReLu activation function in the CBAM attention module. A soybean leaf disease dataset redundancy experiment showed that the CBAM-ConvNeXt model outperformed the other network models in terms of generalization ability and robustness. The average recognition accuracy of the CBAM-ConvNeXt model on the augmented test set was 85.42%, which was higher than that of the other network models under the same experimental conditions. This indicates that the proposed model improvement method has a favorable impact on soybean leaf disease classification.

The model exhibits the lowest performance in detecting leaf diseases that have a high similarity in their essential features. In the future, a combination of small-sample learning methods should be used to improve model performance when using a smaller training data set. In addition, the number of parameters in the model was found to be a gap compared to the lightweight network model in the experiments, which is not conducive to the deployment of the model to mobile, so lightweighting of the model is also an important research direction.

## Data Availability

As the Jilin College of Agricultural Science and Technology “Smart Agriculture” dataset is not yet fully established, the datasets analysed in the current study are not publicly available, but are available on request from the corresponding author, You Tang, upon reasonable request.

## Code availability

The main code can be accessed at the PaddlePaddle platform, the access link is: https://aistudio.baidu.com/projectdetail/5363906?contributionType=1.
